# Latent Inhibition in Schizophrenia and Schizotypy

**DOI:** 10.1093/schizbullopen/sgad026

**Published:** 2023-11-17

**Authors:** Liam Myles, Jane Garrison, Lucy Cheke

**Affiliations:** Department of Psychology, University of Cambridge, Cambridge, UK; Department of Psychology, University of Cambridge, Cambridge, UK; Department of Psychology, University of Cambridge, Cambridge, UK

**Keywords:** Salience Hypothesis, Schizophrenia, Schizotypy, Latent Inhibition, Hallucinations, Unusual Beliefs

## Abstract

**Background:**

The Salience Hypothesis posits that aberrations in the assignment of salience culminate in hallucinations and unusual beliefs, the “positive symptoms” of schizophrenia. Evidence for this comes from studies on latent inhibition (LI), referring to the phenomenon that prior exposure to a stimulus impedes learning about the relationship between that stimulus and an outcome.

**Design:**

This article reviewed all published studies examining the relationship between LI and both schizophrenia and schizotypy.

**Results:**

Contemporary literature suggests that LI is attenuated in both people with schizophrenia and those loading highly on measures of schizotypy, the multidimensional derivative of schizophrenia. This suggests that these individuals assign greater salience to stimuli than healthy controls and people scoring low on measures of schizotypy, respectively. However, several confounds limit these conclusions. Studies on people with schizophrenia are limited by the confounding effects of psychotropic medications, idiosyncratic parsing of samples, variation in dependent variables, and lack of statistical power. Moreover, LI paradigms are limited by the confounding effects of learned irrelevance, conditioned inhibition, negative priming, and novel pop-out effects.

**Conclusions:**

This review concludes with the recommendation that researchers develop novel paradigms that overcome these limitations to evaluate the predictions of the Salience Hypothesis.

## Introduction

### The Salience Hypothesis of Schizophrenia

Schizophrenia is a complex, heterogenous condition, affecting approximately 1% of people.^1,2^ Historically, symptoms have been divided into “positive,” “negative,” and “disorganized.”^3–6^ Positive symptoms refer to hallucinations, unusual beliefs (so-called "delusions"), and disorganized thoughts, whereas negative symptoms concern social withdrawal and reductions in motivation, emotional affect, pleasure, and speech.^7^ Cognitive difficulties can also be experienced, including deficits in attention, decision making, memory, and planning.^7^

Although they possess some utility, research suggests that psychiatric diagnoses, such as schizophrenia, lack robust evidence as qualitatively distinct phenomena from the experiences of the general population.^8–11^ Thus, where possible, a multidimensional view of schizophrenia will be adopted, acknowledging that symptoms are distributed throughout the population to varying degrees.^12–15^ Where diagnostic terms are used, these refer to “people that have received that diagnosis.”

Since the inception of the diagnosis,^16^ it has been suggested that attention is disrupted in people with schizophrenia.^17–24^ Qualitative accounts indicate that perceptual sensations can be perceived to be “heightened” and familiar stimuli can appear highly salient and “important.”^25–32^ These accounts contributed to the development of the *Salience Hypothesis*, which suggests that individuals with schizophrenia assign aberrant salience to stimuli, resulting in redundant stimuli maintaining a greater sense of novelty and significance.^29,33,34^ This theory argues that hallucinations occur consequential to this aberrant assignment of salience to internal (self-generated) stimuli and that unusual beliefs arise as one attempts to comprehend and explain the resulting experiences. For example, an individual’s thoughts may be excessively salient such that they are perceived to have an external origin, resulting in the hallucinatory percept of a voice. Unusual beliefs might arise as individuals attempt to rationalize this experience (eg, hearing a voice) to make sense of the world. As unusual beliefs are generated within the context of pre-existing beliefs and schemata, individual differences in presuppositions influence the nature of beliefs derived from aberrantly salient experiences.^35^

### Latent Inhibition

Numerous experimental paradigms have been used to evaluate the Salience Hypothesis’ proposition that aberrant salience is assigned to stimuli, including those leveraging latent inhibition (LI). LI refers to the phenomenon that pre-exposure to a conditioned stimulus (CS) in the absence of an unconditioned stimulus (US) impairs subsequent learning about the CS-US relationship.^36^ Whilst LI had long been established in animals,^36^ seminal evidence that humans exhibit LI came from Ginton et al, who presented control participants with a series of nonsense syllables and instructed them to count the frequency with which they were repeated.^37^ The experimental group underwent an identical procedure, but with the addition of superimposed white noises. Following this, all participants were exposed to nonsense syllables with white noises superimposed, and asked to press a button whenever they heard the stimulus that they believed predicted an increase in points on a scoreboard. Here, the white noise represented the CS, whereas the change in the value of the scoreboard corresponded to the US. Participants learnt the association between the white noise and the change in the value of the scoreboard more quickly in the control group than in the experimental group, indicating that CS pre-exposure impeded learning about CS-US associations. Similar studies have directly and conceptually replicated these results across numerous sensory modalities,^38–40^ suggesting that LI represents a robust cross-modal phenomenon.

Whilst alternative accounts have been proposed,^41^ contemporary theories suggest the CS’s salience declines during pre-exposure, as it fails to predict the US with greater consistency than contextual information, reducing its associability in the conditioning phase.^42–50^ Accordingly, researchers have used LI paradigms to examine the Salience Hypothesis’ assumption that people with schizophrenia assign salience aberrantly. The remaining sections of this article aim to provide a ­narrative review of all English-written, published studies examining the effect of schizophrenia and/or schizotypy on LI.

### Schizophrenia and Latent Inhibition

Although there are inconsistencies, research suggests that LI may be disrupted in individuals with schizophrenia. Despite early studies indicating that LI is intact,^51^ Baruch et al ascertained seminal evidence that auditory LI is disrupted in individuals with schizophrenia, using Ginton et al’s paradigm.^52^ This study differentiated between “chronic” and “acute” schizophrenia, referring to people with schizophrenia for more or less than 6 months, respectively. While controls and people with chronic schizophrenia exhibited LI, CS pre-exposure failed to impede learning in participants with acute schizophrenia. However, replication studies have produced inconsistent support for the finding that LI impairment is greater at shorter, compared with longer, latencies after the onset of schizophrenia.^53,54^ Indeed, longitudinal evidence for temporal-based variations in LI in people with schizophrenia is, to the best of the author’s knowledge, absent, making it unclear whether chronicity is causally associated with LI. Nevertheless, subsequent studies have reported that people with schizophrenia exhibit attenuated LI across auditory^55,56^ and visual modalities,^57^ supporting the notion that the salience of redundant stimuli is elevated.^44,58^

There is some, albeit limited, evidence that LI is also attenuated in other modalities. For example, electrodermal LI, corresponding to reduced skin conductance responses to visual CSs after pre-exposure, has been reported to be attenuated in unmedicated people with schizophrenia.^54^ However, reduced electrodermal responding in the pre-exposed group may also reflect habituation to the CS.^59^ Thus, schizophrenia may be associated with attenuated electrodermal habituation, rather than LI. Similar results have been ascertained using neurophysiological measurements, with reports that CS pre-exposure delays the build-up of contingent negative variation (CNV), an electroencephalographic measure of associative learning,^58,60,61^ in controls but not medicated individuals with schizophrenia.^62^ Whilst this was interpreted as evidence that schizophrenia impairs the direction of attention toward relevant stimuli, this study did not ascertain behavioral evidence of LI in both patients and controls, casting doubt on the proposition that CNV measured LI in this study. Indeed, replication studies failed to find evidence that CNV is impaired in individuals with schizophrenia.^63^ Therefore, these results provide only tentative evidence that LI is attenuated across electrodermal and neurophysiological measurement modalities in those with schizophrenia.

Many of the aforementioned studies are limited by the use of between-subjects designs that compare learning in pre-exposed and non–pre-exposed groups.^52,55,62^ Between-subjects designs can introduce systematic confounds, such as the relative novelty of the stimuli at test for the non–pre-exposed, but not the pre-exposed, group.^64^ Also, matching groups on key demographic variables, a necessary precaution to control for systematic confounds, is challenging in patients and rarely conducted appropriately.^65^ These confounds may artificially inflate between-group differences.

Using a within-subjects design, in which points on a scoreboard were contingent upon both a non–pre-exposed tone and pre-exposed white noise, Gray et al reported that unmedicated people with schizophrenia exhibited intact LI.^53^ While these results contradict the Salience Hypothesis, the authors did not conduct the necessary interaction test to examine whether the effect of pre-exposure varied between patients and controls. Moreover, they did not counterbalance stimuli, rendering the results amenable to the explanation that the salience of the white noise was lower than the tone.^49^

More convincing within-subjects evidence comes from studies relating LI to symptomology. Research indicates that the extent of LI attenuation is correlated with the severity of positive symptoms in unmedicated people with schizophrenia.^66^ Interestingly, people with acute, compared with chronic, schizophrenia exhibit greater positive symptoms^67^; this may account for demonstrations that the former exhibit greater LI deficits than the latter.^52,53,55,68^ Moreover, there is tentative evidence that *potentiated* LI, corresponding to even greater reductions in learning consequential to pre-exposure, is associated with negative symptom severity.^56^ In a sample of predominantly medicated people with schizophrenia, Cohen et al reported that participants with a combination of high negative and low positive symptoms exhibited potentiated visual LI, compared with those with other clusters of symptoms.^67^ However, parsing participants by symptom clusters resulted in underpowered groups, with only 6 participants in the group displaying potentiated LI, necessitating caution in interpreting these findings. Similar studies have found inconsistent results, with limited evidence that positive symptoms moderate the relationship between negative symptoms and LI.^56^ Therefore, whilst there is some evidence that positive symptoms are associated with LI attenuation, the relationship with negative symptoms remains unclear.

However, numerous studies have reported intact auditory and visual LI in medicated people with schizophrenia.^69,70^ Nevertheless, whilst Swerdlow et al reported that patients exhibited significantly poorer learning in the pre-exposed condition,^69^ re-computation of the *P* values^71,72^ indicates that such effects did not meet conventional thresholds for statistical significance (*P* = .08). Also, patients exhibited inferior learning in the non-pre-exposed condition in both studies, suggesting performance was confounded by general deficits in associative learning. Indeed, a disproportionally high number of patients failed to learn the CS-US contingency, compared with controls; participants that failed to learn the association may have exhibited impaired LI, but remained undetected. Therefore, whilst the Salience Hypothesis garners support from studies suggesting that individuals with schizophrenia exhibit cross-modal attenuations in LI and that this disruption is related to positive symptoms, inconsistencies in the literature challenge the reliability of these results. Studies examining the relationship between schizophrenia and LI are summarized in table 1.

**Table 1.** Studies Examining the Relationship Between Schizophrenia and Latent Inhibition

Authors	N		Design	Stimulus Modality	DV	Results
	P	C				
Baruch et al. (1988a)	53	53	Between subjects	Auditory	TTC	LI was impaired in people with “acute,” but not “chronic,” schizophrenia.
Cohen et al. (2004)	30	30	Within subjects	Visual	RT	Participants with high negative and low positive symptoms exhibited potentiated visual LI, compared with those with other clusters of symptoms.
Gal et al. (2009)	19	20	Within subjects	Visual	RT	Participants with schizophrenia showed attenuated LI in the first 5 test trials and potentiated LI in the second 5 test trials.
Gray et al. (1995)	15	13	Within subjects	Auditory	TTC	LI was intact in people with schizophrenia.
Guterman et al. (1996)	14	14	Between subjects	Auditory	CNV	CNV build-up was delayed in controls, but not people with schizophrenia, suggesting LI was impaired.
Kathmann et al. (2000)	33	20	Between subjects	Auditory	CNV and ERP	CNV build-up was intact in people with schizophrenia, suggesting LI was intact.
Lubow et al. (1987)	39	48	Between subjects	Auditory	TTC	LI was intact in people with schizophrenia.
Rascle et al. (2001)	65	40	Between subjects	Auditory and visual	TTC	LI was attenuated in people with acute schizophrenia and potentiated in those with chronic schizophrenia.
Serra et al. (2001)	21	24	Between subjects	Auditory	TTC	LI was “attenuated” in people with schizophrenia, due to impaired learning in the non-pre-exposed group.
Swerdlow et al. (1996)	45	73	Between subjects	Auditory and visual	TTC	Auditory and visual LI were intact in medicated people with schizophrenia.
Swerdlow et al. (2005)	38	60	Within subjects	Visual	A	LI was intact in people with schizophrenia.
Vaitl et al. (2002)	32	16	Within subjects	Visual	SCR	Electrodermal LI was impaired in medicated, but not unmedicated, people with schizophrenia.
Williams et al. (1998)	102	73	Between subjects	Auditory		LI was attenuated in medicated, but not unmedicated, people with schizophrenia.
Yogev et al. (2004)	41	24	Between subjects	Auditory	TTC	LI attenuation is correlated with positive symptom severity in unmedicated people with schizophrenia.

*Note*: In all tables, “*N*” refers to the number of participants; “P” corresponds to patients with schizophrenia, whereas “C” refers to controls. “DV” pertains to the dependent variable and consisted of either accuracy (A), contingent negative variation, event-related potentials (ERPs), reaction time (RT), skin conductance response (SCR), or trials-to-criterion (TTC). CNV, contingent negative variation; LI, latent inhibition.

### Limitations of Studying Participants With Schizophrenia

Several limitations cast doubt on the validity and reliability of LI studies using participants with schizophrenia. Firstly, the use of psychotropic medication confounded many of these studies. LI can be enhanced in people with and without schizophrenia after taking antipsychotic medications,^54,73–77^ suggesting that psychotropic medications may “normalize” attentional processes.^78,79^ Therefore, demonstrations that people with “acute,” but not “chronic,” schizophrenia show attenuated LI^52,55^ may stem from the greater likelihood of the latter to be taking psychotropic medications.^67^ Also, reports that negative symptoms are associated with potentiated LI^56,67^ may be an artifact of the propensity of psychotropic medications to increase negative symptoms.^80–82^

Secondly, the segmentation of patients by symptom longevity or demographic variables, without sufficient empirical basis, inflates the risk of Type I errors.^83,84^ For example, the temporal-based distinction between “acute” and “chronic” schizophrenia has little basis in empirical evidence^8–11^ and varies between studies.^52,55,56^ This is further hindered by challenges in determining when symptoms commenced due to the common delay between symptom onset and contact with psychiatric services, particularly in the era in which these studies were conducted.^85–87^ Moreover, subgroups of patients that display “impaired LI” often exhibit poorer learning in the non-pre-exposed condition,^53,54,63,88–90^ rendering results attributable to general learning deficits. The absence of empirical justifications for the crude and variable distinctions between subgroups, and the common necessity for idiosyncratic parsing of data to achieve “statistically significant” results,^91^ risks overfitting models to data, casting doubt on the reliability of these results.

Thirdly, the use of different dependent variables, including trials to a specified criterion assumed to indicate “learning” (eg, correct responses on 5 consecutive trials), reaction times (RTs) and accuracy rates, as well as different criteria to signify “learning,” complicates drawing comparisons between studies. It is consequently difficult to determine whether disparate results are theoretically meaningful or artifacts of variations in the dependent variable. There are some practical reasons to prefer measuring RTs to accuracy. A larger proportion of patients than controls fail to learn the CS-US contingency, irrespective of pre-exposure condition, resulting in a bimodal distribution of scores. These floor effects risk occluding differences in learning between the pre-exposed and non–pre-exposed conditions, inflating the probability of observing null results in patients. Whilst measuring RTs can circumvent this issue by producing normally distributed scores, it cannot be considered an equivalent variable. Some studies failed to demonstrate LI in RTs but reported LI when examining trials-to-criterion,^92^ whereas others have demonstrated the reverse trend.^93,94^ These inconsistencies cast doubt on the proposition that trials-to-criterion and RTs examine the same construct, despite their frequent interchangeable use as dependent variables.

Finally, many of the aforementioned studies are underpowered,^95^ particularly those using between-subjects designs. Small samples and the frequent use of nonparametric tests inflate the probability of Type II errors and may occlude differences in learning between the pre-exposed and non–pre-exposed conditions.^92,96^ This is particularly problematic, as demonstrating attenuated LI is often dependent on a null effect in the patient group. Given the chronic lack of statistical power, the vast array of conflicting results is not only unsurprising, but expected. These limitations cast doubt on the validity and reliability of LI studies on people with schizophrenia.

### Schizotypy and Latent Inhibition

Contemporary theories advocate a multidimensional conceptualization of schizophrenia, acknowledging that positive, negative, and disorganized symptoms are distributed throughout the population to varying degrees.^12–14,97^ These characteristics, termed “schizotypal traits,”^98–101^ vary on dimensions of “unusual experiences,” “introvertive anhedonia,” “cognitive disorganization,” and “impulsive non-conformity,” corresponding to the dimensional derivatives of positive symptoms, negative symptoms, disorganized symptoms, and impulsive behavior with low self-control, respectively.^102,103^ Factor analytic studies indicate that schizophrenia and schizotypy maintain similar factor structures^102,104–111^ and longitudinal evidence suggests that elevated schizotypy is associated with vulnerability to schizophrenia,^107,112–114^ supporting the notion that schizotypy represents the multidimensional derivative of schizophrenia. Accordingly, researchers have examined the relationship between schizotypy and LI, to inform theories of schizophrenia whilst overcoming the limitations of studying patients.

Whilst there are inconsistencies, research indicates that LI is attenuated in high schizotypy individuals. Seminal evidence comes from Baruch et al, who, using Ginton et al’s auditory paradigm, reported that learning in the pre-exposed condition was greater in people scoring high, compared with low, on the Eysenck Personality Questionnaire Psychoticism Scale (EPQ-P).^115,116^ Replicating these results, Lubow et al reported that attenuated LI was associated with high scores on both the EPQ-P or Schizotypal Traits Questionnaire (STQ).^117,118^ However, the relationship between LI and the STQ was attributable to variations in learning in the non–pre-exposed group, suggesting that participants scoring highly on this measure exhibited general learning impairments. More convincingly, in a large sample of 205 participants, Allan et al reported that auditory LI^37^ was attenuated in high, but not low, scorers on both the EPQ-P and the STQ, consequential to potentiated learning in the pre-exposed condition.^119^ Indeed, these results have been repeatedly replicated.^38,89^

As with schizophrenia, the attenuation of LI in high schizotypy participants extends to other modalities, including vision. Whilst Lubow et al initially reported intact visual LI in high schizotypy participants, they did not statistically examine whether variations in LI were attributable to differences in the pre-exposed or non–pre-exposed group, rendering the results difficult to interpret.^118^ Subsequent research has repeatedly demonstrated that high schizotypy participants exhibit attenuated visual LI, consequential to greater learning in the pre-exposed condition, across various measures of schizotypy, including the EPQ-P,^120,121^ Schizotypal Personality Questionnaire (SPQ),^122–124^ STQ,^94,118,121,125–128^ and the Multiphasic Personality Inventory^129,130^; analogous results were ascertained in a study of 247 youths.^131^ Interestingly, Casa et al demonstrated that high schizotypy participants exhibit visual LI, but only after extensive CS pre-exposure.^130^ These results provide an important addendum to the Salience Hypothesis, suggesting that the salience of redundant stimuli may take longer to wane, rather than remaining high indefinitely, in high schizotypy individuals. Indeed, whilst studies indicate that schizotypy does not influence the perceived salience of stimuli,^127^ as determined by performance on a “novel pop-out” paradigm,^132,133^ high schizotypy participants exhibit greater recognition of previously presented stimuli.^130,134^ This provides tentative evidence that high, compared with low, schizotypy individuals may maintain attention to stimuli for longer durations, resulting in deeper processing and consequently superior recognition.^135,136^

Similar results have been reported in the electrodermal measurement modality, though this evidence is more mixed. For example, studies suggest electrodermal LI is present in low, but not high, schizotypy participants.^137^ Whilst this relationship was only observed when measuring schizotypy with the STQ, and not the EPQ-P or Launay-Slade Hallucination Scale (LSHS),^138^ subsequent studies have replicated these findings using both the SPQ or LSHS.^90,139^ However, replication was dependent upon theoretically arbitrary factors, such as the hand from which electrodermal measurements were recorded.^90^ The authors did not correct for multiple comparisons when splitting the data with respect to the participant’s hand and, had they applied the Bonferroni correction, group differences would not have reached conventional thresholds for statistical significance.^95^ Also, they did not report whether there was an interaction between schizotypy status and pre-exposure condition, limiting the conclusion that LI differed with respect to schizotypy. Thus, whilst there is preliminary evidence of an association between schizotypy and electrodermal LI, future research is required to clarify the nature of this relationship.

Contemporary literature suggests that LI attenuation may be specifically associated with unusual experiences, a dimension often measured by the Oxford-Liverpool Inventory of Feelings and Experiences (O-LIFE). Using a visual paradigm, Burch et al reported that individuals scoring high, but not low, on unusual experiences displayed attenuated LI^140^; unfortunately, the authors did not conduct the appropriate post hoc tests to statistically confirm this. Replication studies support the conclusion that unusual experiences are associated with attenuated visual LI, consequential to greater learning in the pre-exposed condition.^68,141,142^ However, re-computation of the *P* values^71,72^ in Gray et al’s study indicates that the relationship between unusual experiences and learning in the pre-exposed group failed to meet conventional thresholds for statistical significance (*P* = .09). Nevertheless, these findings suggest that unusual experiences may be associated with attenuated LI, paralleling the relationship between LI and positive symptoms in schizophrenia.

Despite these compelling findings, several studies have reported contradictory findings of intact LI in high schizotypy individuals,^69,125,139,141^ or failed to demonstrate attenuated LI without parsing the sample in idiosyncratic ways.^121^ These discrepant results may partially stem from the use of the “median-split method” in all but one^127^ of the studies discussed thus far, in which “high” and “low” schizotypy participants are defined by schizotypy scores above and below the median, respectively. Idiosyncratic differences in the median scores, due to sample variation, and schizotypy measures used complicates drawing comparisons between studies. Also, heterogeneity in scores within groups may occlude differences in LI between groups. These limitations may partially account for contradictory results.

Another limitation of LI paradigms concerns the use of “masking tasks,” which entail completing a concurrent task during pre-exposure. Evidence that LI represents an attentional phenomenon originated in animal studies^44^ and masking tasks are used in humans to promote automatic, rather than controlled, processing of stimuli to parallel the “low level” attentional processes assumed, perhaps incorrectly,^143^ to be used by animals.^39,58^ However, whilst masking tasks are often required to produce both visual^144^ and auditory LI,^145^ they are not used in animal studies,^146^ arousing concern that performance in human and animal LI paradigms is dependent on distinct cognitive processes.^143^ This underscores the necessity to demonstrate LI without masking tasks.

More recent studies have examined LI using multiple regression, rather than the median-split method, and without masking tasks. Using a within-subjects paradigm, Evans et al demonstrated that people higher in unusual experiences exhibited a trend toward faster (*P* < .08) and more accurate (*P* = .10) learning in the pre-exposed, but not the non-pre-exposed, condition of a visual LI paradigm.^65^ Replication studies found clearer evidence that participants higher in unusual experiences exhibited significantly faster and more accurate (*P* < .05) learning in the pre-exposed, but not the non-pre-exposed, condition^147,148^; however, Chun et al’s replication found null results using the Wisconsin Schizotypy Scales.^149,150^ Overall, these results suggest that high schizotypy individuals exhibit cross-modal attenuations in LI and that this disruption is selectively associated with unusual experiences, in line with research on schizophrenia and the predictions of the Salience Hypothesis. Studies examining the relationship between schizotypy and LI are summarized in table 2.

**Table 2.** Studies Examining the Relationship Between Schizotypy and Latent Inhibition

Authors	*N*	Design	Stimulus Modality	DV	Results
Allan et al. (1995)	205	Between subjects	Visual	TTC	LI was attenuated in high, compared with low, schizotypy participants.
Baruch et al. (1988b)	53	Between subjects	Auditory	TTC	LI was attenuated in high, compared with low, schizotypy participants.
Braunstein-Bercovitz (2000)	60	Between subjects	Visual	A and RT	LI was attenuated in high, compared with low, schizotypy participants. However, this was attributable to differences in both the pre-exposed and non-pre-exposed groups.
Braunstein-Bercovitz (2003)	58	Between subjects	Visual	A and RT	LI was attenuated in high, compared with low, schizotypy participants. However, this was attributable to differences in both the pre-exposed and non-pre-exposed groups.
Burch et al. (2004)	80	Between subjects	Visual and auditory	TTC	Visual LI was attenuated in participants scoring high, compared with low, in unusual experiences. Auditory LI was intact in participants high in unusual experiences.
Burch et al. (2006)	100	Within subjects	Visual	RT	LI was attenuated in those high, compared with low, in unusual experiences. However, these effects appeared to stem from differences in the non-pre-exposed, but not the pre-exposed, group; statistical evidence for this speculation is absent.
Chun et al. (2019)	84	Within subjects	Visual	RT	LI was intact in high schizotypy participants.
Dawes et al. (2022)	346	Within subjects	Visual	RT	LI was intact in high schizotypy participants.
De la Casa et al. (1993)	120	Between subjects	Visual	TTC	LI was attenuated in high, compared with low, schizotypy participants.
De la Casa et al. (1999)	105 and 102	Between subjects	Auditory and visual	TTC	Visual LI was attenuated in high, compared with low, schizotypy participants after 10 pre-exposures; auditory LI did not vary with schizotypy. After 30 pre-exposures, schizotypy was not associated with visual or auditory LI.
Evans et al. (2007)	80	Within subjects	Visual	A and RT	Those higher in unusual experiences exhibited a trend toward faster (*P* < .08) and more accurate (*P* = .10) learning in the pre-exposed, but not the non-pre-exposed, condition.
Granger et al. (2012)	33 and 34	Within and between subjects	Visual	RT	LI was attenuated in participants high, compared with low, in unusual experiences.
Granger et al. (2016)	57 and 60	Within subjects	Visual	RT	LI was potentiated in participants high, compared with low, in unusual experiences.
Gray et al. (2002)	80	Between subjects	Visual	TTC	LI was attenuated in individuals high in cognitive disorganization and impulsive non-conformity.
Gray et al. (2003)	96	Within subjects	Auditory	TTC	LI was attenuated in high, compared with low, schizotypy participants.
Höfer et al. (1999)	101	Between subjects	Visual	TTC	LI was attenuated in high, compared with low, schizotypy participants.
Kraus et al. (2016)	247	Within subjects	Visual	A and RT	LI was attenuated in participants “ultra-high-risk” for psychosis.
Lipp and Vaitl (1992)	48	Between subjects	Visual	SCR	Electrodermal LI was attenuated in high, compared with low, schizotypy participants, as measured by the STQ but not the EPQ or LSHS.
Lipp et al. (1994)	76	Between subjects	Visual	SCR	Electrodermal LI was attenuated in high, compared with low, schizotypy participants.
Lubow and De la Casa (2002)	12 and 44	Between subjects	Visual	A and RT	LI was attenuated in high, compared with low, schizotypy participants, as measured by RTs but not accuracy. In a replication, LI was attenuated in high, compared with low, schizotypy females, as measured by RTs and accuracy.
Lubow et al. (1992)	48	Between subjects	Auditory	TTC	“LI” was attenuated in high, compared with low, schizotypy participants; however, this effect was attributable to differences in learning in the non-pre-exposed group.
Lubow et al. (2001)	180	Within subjects	Visual	RT	LI was attenuated in high, compared with low, schizotypy participants; however, this was only observed for females.
Serra et al. (2001)	22	Between subjects	Auditory	TTC	LI was attenuated in high, compared with low, schizotypy participants.
Shrira and Tsakanikos (2009)	115	Between subjects	Visual	A	LI was attenuated in people high in unusual experiences and potentiated in people high in introvertive anhedonia.
Schmidt-Hansen et al. (2009)	22	Between subjects	Visual	A and RT	LI, as measured by accuracy and RTs, was attenuated in people high in unusual experiences in block 2, but not block 1; the authors failed to replicate this finding in a similar study.
Swerdlow et al. (1996)	73	Between subjects	Auditory	TTC	LI was intact in individuals classified as “psychosis-prone.”
Tsakanikos (2004)	32	Between subjects	Visual	A	LI was attenuated in high, compared with low, schizotypy participants.
Tsakanikos and Reed (2004)	60	Between subjects	Visual	A	LI was attenuated in high, compared with low, schizotypy participants.

*Note*: DV, dependent variable; EPQ, Eysenck Personality Questionnaire; LI, latent inhibition; LSHS, Launay-Slade Hallucination Scale; RT, reaction time; SCR, skin conductance response; STQ, Schizotypal Traits Questionnaire; TTC, trials-to-criterion.

### Limitations of Latent Inhibition Paradigms

Whilst LI paradigms have been informative in the study of attention in schizophrenia, these tasks are subject to alternative accounts.

*Learned irrelevance* refers to learning that a CS actively *does not* predict a US after uncorrelated exposures to the CS and US. This is distinct from LI, as both the CS and US are presented during pre-exposure. Nonetheless, human LI paradigms are confounded by learned irrelevance. Specifically, as the CS is irrelevant to the masking task, participants may learn that the CS is irrelevant altogether, impeding learning in the pre-exposed group.^151^ Therefore, high schizotypy participants may experience impaired learned irrelevance, rather than LI. Indeed, there is evidence that learned irrelevance is reduced in high, compared with low, schizotypy participants,^152^ and that the extent of this impairment is correlated with unusual experiences^148,153^; analogous results have been reported in people with schizophrenia.^57,154,155^

*Conditioned inhibition*, learning that a CS signals the absence of a US, also confounds LI paradigms that avoid using masking tasks. For example, Evans et al instructed participants to press a button when they saw a letter that they believed predicted the occurrence of the letter “X”; participants were slower to learn the association between X and a pre-exposed, compared with a non-pre-exposed, letter.^65^ Pre-exposing stimuli in the absence of X, which participants knew was the target, may have resulted in the pre-exposed stimulus forming an inhibitory association with X, as it consistently predicted the absence of X during pre-exposure.^156^ Moreover, averaged across pre-exposure and test trials, the CS-US contingency was lower in the pre-exposed than the non–pre-exposed condition.^95^ Therefore, “LI” may reflect accurate learning that the CS-US contingency is lower across trials, and it may be a tendency to “average” the associability of the CS across trials, rather than utilizing information from only the test phase, to determine the CS-US contingency that is reduced in high schizotypy participants.^64^ However, despite human LI procedures seemingly encouraging participants to integrate information regarding CS-US associability across trials,^95^ manipulating the likelihood of integrating information, through alterations to or removal of instructions, yields minimal influence over LI.^128,157,158^ This offers some reassurance that instructions maintain a minimal effect on LI, and thus that conditioned inhibition does not, at least entirely, underpin “LI effects.”

To overcome these confounds, Granger et al conducted a similar paradigm to Evans et al,^65^ but participants were not informed of the target, eliminating the potential for conditioned inhibition, and were required to state each letter aloud during pre-exposure, minimizing learned irrelevance by establishing each letter as task-relevant.^159^ Paradoxically, greater unusual experiences were associated with *enhanced* LI, consequential to slower learning in the pre-exposed group. These results contradict the aforementioned literature, implying that conditioned inhibition and learned irrelevance may indeed have produced an apparent relationship between unusual experiences and “attenuated LI” in prior studies. However, using a different schizotypy questionnaire, Dawes et al failed to find any association between performance on this task and SPQ scores.^160^

Another limitation concerns the potential for LI paradigms to generate *negative priming* effects.^143^ During pre-exposure, the masking task and CS represent the target and distractor stimuli, respectively, in the pre-exposed group; at test, this is reversed. In the non–pre-exposed group, whilst participants must ignore the previously relevant masking stimuli at test, the target is novel. The reversal of target and distractor stimuli may impair learning in the pre-exposed group, rather than the declination of the CS’s salience and consequential associability.^96,146^ Therefore, “attenuated LI” in high schizotypy participants may represent deficits in negative priming.

Finally, whilst stimuli are invariant across pre-exposure and test phases in the pre-exposed group, a novel stimulus is introduced at test in the non–pre-exposed group. The “novel pop-out effect” refers to the phenomenon that novel stimuli are identified more readily when placed on a background of familiar distractors.^161,162^ Accordingly, in LI paradigms, the non–pre-exposed group may exhibit greater learning due to familiar distractors enhancing the salience of the novel CS at test; this would imply that LI is a phenomenon of the non–pre-exposed, rather than pre-exposed, condition.^88^ These limitations do not invalidate LI as a concept but instead render results from human LI studies contentious, highlighting the necessity for novel paradigms that overcome these limitations.

### Clinical Implications

This review entails several clinical implications. Examining whether LI is attenuated in people with schizophrenia provides greater insight into the psychological processes underpinning hallucinations and unusual beliefs. Contemporary approaches to conceptualizing psychiatric conditions are increasingly focusing on “transdiagnostic processes.”^163–165^ Understanding the learning mechanisms that influence perception and beliefs may shed light on the causal basis of psychological conditions and, critically, potential interventions for people struggling with or at risk of developing such conditions.^166,167^

Also, this review provides avenues for future research which may ultimately result in the development of interventions for people with schizophrenia. It would be inappropriate to conclude that variations in LI entail a causal role in either schizophrenia or schizotypy based on current literature. However, if future research ascertains robust evidence of causality, interventions aimed at altering LI may support individuals to reduce distressing positive symptoms. For example, pharmacological interventions may be used to alter LI^54,73–77,168^ to support people with schizophrenia. Nevertheless, studies examining whether LI maintains a causal role in both schizophrenia and schizotypy are necessary before corresponding interventions can be developed.

### Limitations

Whilst these results have been interpreted within a salience-based account of LI, other psychological processes may underpin LI. For example, LI may stem from the formation of “CS-no US” associations, referring to excitatory associations between CSs and the absence of outcomes.^169^ Accordingly, pre-exposure may result in strong CS-no US associations, and both schizophrenia and schizotypy may be associated with variations in the formation of these, rather than stimulus salience.^170^ Moreover, strong evidence suggests that LI is context specific^39,171^ and may depend on the extent to which the context associatively primes the CS.^50^ As research indicates that context processing is impaired in high schizotypy individuals,^21,172,173^ the context may be less able to associatively prime pre-exposed stimuli, resulting in attenuated LI. Alternatively, as transitions between pre-exposure and test phases may be perceived as context changes, perhaps due to changes in instructions, associations between LI and both schizophrenia and schizotypy may reflect variations in the extent to which the test phase is perceived as a novel context. For example, if the test phase is treated as a novel context, it will be less able to associatively prime stimuli, impairing LI.^146^ Further research is necessary to confirm the mechanisms underpinning LI and their relationship with both schizophrenia and schizotypy.

### Future Research

The limitations associated with current LI paradigms underscore the necessity to develop novel paradigms to examine LI in people with schizophrenia and high schizotypy individuals. One possible alternative is paradigms examining perceptual learning (PL), the phenomenon that pre-exposing participants to 2 highly similar stimuli aids discrimination between the stimuli.^174–181^ Whilst a detailed explanation of PL is beyond the scope of this article, contemporary theory argues that LI of the features common to both stimuli underpins this phenomenon.^182,183^ Critically, PL paradigms circumvent confounds of learning irrelevance, conditioned inhibition, and negative priming because USs and masking tasks are not presented during pre-exposure. Also, novel pop-out effects do not confound these paradigms, as stimuli are identical during pre-exposure and test phases. Future research may benefit from examining the effect of both schizophrenia and schizotypy on PL, to corroborate the claim that LI is attenuated in people with schizophrenia.

## Conclusion

The Salience Hypothesis represents a promising account of the psychological mechanisms underpinning the positive symptoms of schizophrenia, and some of the strongest supportive evidence comes from LI studies. Indeed, there is evidence that positive symptoms are associated with attenuated LI. However, this research is limited by the confounding effects of psychotropic medications, idiosyncratic parsing of samples, variation in dependent variables, and lack of statistical power. Research suggests that non-diagnosed individuals high in unusual experiences also exhibit attenuated LI, but these studies are limited by the confounding effects of learned irrelevance, conditioned inhibition, negative priming, and novel pop-out effects. Elucidating whether the attribution of salience is disrupted in people with schizophrenia is essential, both to inform theoretical accounts of hallucinations and unusual beliefs and to improve clinical interventions for this population. The issues associated with LI paradigms highlight the necessity for researchers to develop novel paradigms that overcome these limitations to examine whether the attribution of salience is disrupted in people with schizophrenia and high schizotypy individuals.
